# The Role of Immunosenescence in Neurodegenerative Diseases

**DOI:** 10.1155/2018/6039171

**Published:** 2018-03-08

**Authors:** Erica Costantini, Chiara D'Angelo, Marcella Reale

**Affiliations:** Unit of Immunodiagnostic and Molecular Pathology, Department of Medical, Oral and Biotechnological Sciences, “G. d'Annunzio” University of Chieti-Pescara, 66100 Chieti, Italy

## Abstract

Aging is characterized by the progressive decline of physiological function and tissue homeostasis leading to increased vulnerability, degeneration, and death. Aging-related changes of the innate and adaptive immune system include decline in the preservation and enhancement of many immune functions, such as changes in the number of circulating monocytic and dendritic cells, thymic involution, T cell polyfunctionality, or production of proinflammatory cytokines, and are defined as immunosenescence. Inflammatory functions are increased with age, causing the chronic low-grade inflammation, referred to as inflamm-aging, that contribute, together with immunosenescence, to neurodegenerative diseases. In this review, we discuss the link between the immune and nervous systems and how the immunosenescence and inflamm-aging can contribute to neurodegenerative diseases.

## 1. Immunosenescence

In the last century, the human lifespan has increased and so has the number of elderly people in the world. Aging is a complex process that occurs in every organism and is induced by genetic, epigenetic, and environmental factors [1]. It is characterized by changes at the molecular, cellular, and tissue levels [2]. The immune system is responsible for defending against pathogens such as bacteria, viruses, and fungi to eliminate broken and harmful cells, like senescent cells and toxic or allergenic substances [3]. In the immune system, there is an innate compartment, consisting of neutrophils monocytes/macrophages, natural killer (NK) cells, and dendritic cells (DC), and an adaptive compartment, composed of B and T lymphocytes, which have a well-orchestrated interaction. Immunosenescence is a term that describes a different state of the immune system in aged people, in association with detrimental clinical outcome, due to reduced ability to respond to new antigens [4]. Although immunosenescence is a phenomenon present in the majority of individuals, factors like genetic, environment, lifestyle, and nutrition are responsible for their heterogeneity among individuals and cause a higher susceptibility to develop infections and progression of disease pathology [5]. Studies on immunosenescence have been performed in vitro in human-derived cell lines, and in vivo in animal models, to evaluate their response to different stimuli. Furthermore, the age-related dysregulation of immune responses impacts the resistance to infections, diminishes responses to vaccination, increases the susceptibility to autoimmunity and cancer [6], and promotes the development of an inflammatory phenotype [7]. Franceschi et al. [8] have introduced the term “inflamm-aging ”, related to the immunosenescence, to describe a low-grade, asymptomatic, chronic, and systemic inflammation, characterized by increased levels of circulating cytokines and other proinflammatory markers [6, 8, 9]. The relationship between aging and chronic disorders, including atherosclerosis, dementia, neurodegeneration, and many others, has its bases in senescent remodeling of immune system.

Although research is making significant progress, the impact of immunosenescence on the onset and progression of neurodegeneration remains incompletely clarified; in fact, not necessarily being able to favorably modulate the functions of immune cells, which results in a corresponding change in the clinical outcome. This will not be, by any means, a comprehensive review of the immunosenescence; in fact, this review is focused on changes in the immune system relevant to several neurodegenerative diseases.

## 2. Immunosenescence of Innate Immune Response Cells

The cells of the innate immune system form the first barrier against any pathogen. Neutrophils, monocytes/macrophages and DC, and NK are produced during fetal life and are continuously developed throughout the lifetime [10]. Microglia are tissue-resident macrophages in the CNS (central nervous system), derived from the yolk sac during embryogenesis, that colonize the developing brain where they stay during the individual's lifetime and, like macrophages in the periphery, act as the first line of defense. Phenotype and function of cells involved in innate immune response cells are profoundly influenced by aging, as described by Solana et al. [11]. Immunosenescence of the innate immune system has great complexity and seems to reflect dysregulation, rather than only impaired function. In fact, several responses in the innate immune system are reduced with aging, but, in contrast, also an age-associated hyperreactivity of innate immunity may be evidenced.

### 2.1. Neutrophils

They represent the first cells recruited in the presence of damage and during acute inflammation. Neutrophils are able to produce many degradative enzymes, antimicrobial peptides, and reactive oxygen species (ROS) for antibacterial activity. Many studies demonstrated that the activation of neutrophils, the free radical production signals, and the chemotactic ability are reduced in elderly people [7, 11–13].

The literature reports controversial results about the effect of aging on the amount of neutrophils [14] and their altered functionality. Minet-Quinard et al. demonstrated the presence of immature neutrophils, the production of high levels of intracellular reactive oxygen species, and the expression of activation markers such as CD11b and HLA-DR in the whole blood of advanced-age frail elderly [15]. In contrast with these findings, Sauce reported, in 2016, that ROS production by neutrophils is strictly dependent on priming their presence by proinflammatory mediators. At basal conditions, there are no differences between young and older people, but in the presence of a TNF-*α* (tumor necrosis factor) agonist, like bacterial fMLP (formyl peptides) or PMA (phorbol 12-myristate 13-acetate), ROS (reactive oxygen species) production is impaired across the two groups, with reduction in elderly population [16].

Most recently, Bartlett et al. underlined that the alterations of neutrophil functionality may be different between people of the same age. Chemotaxis, for example, which is a detrimental aspect for response in infection, contributing to the increase in proinflammatory insults persistence, could be positively editable by physical activity in older adults [17].

### 2.2. Macrophages

Monocytes play an important role as starters of the inflammatory response, and they can differentiate into macrophages, antigen-presenting cells, and dendritic cells [6, 12], although more complex differentiation pathways and cell origins have been proposed recently [18]. Macrophages that respond to inflammation stimuli may show two different phenotypes, the classical (M1) and the alternative (M2), depending on the local microenvironment [11]. M1 and M2 are balanced in healthy people, but in the presence of chronic inflammation, as in the presence of inflamm-aging, there is an imbalance, contributing to comorbidities and age-related disease development [19]. Franceschi et al. proposed macrophages as key cells in the induction and maintenance of inflamm-aging and have defined “macroph-aging” as the chronic macrophage activation that characterizes aging [8]. Macroph-aging and inflamm-aging happen in association with immunosenescence, and they reduce efficacy of immune cell activity. Accumulation of senescent macrophages contributes to the acceleration of the aging processes, and conflicting results on macrophage phagocytic function during aging have been reported [20, 21].

Many reports underline how the M2 phenotype develops in the spleen, retina, lymph nodes, and bone marrow in old mice compared to young mice, with higher production of IL- (interleukin-) 10 and reduced production of TNF- (tumor necrosis factor-) *α* [22]. Furthermore, in aged people, the expression of macrophage receptors, such as MHC (major histocompatibility complex) II and TLR (toll-like receptor), is declined with alteration of related activation mechanisms. In both human [11] and murine [23] models, a reduction of MHC II molecules expression was demonstrated, with decline in the ability to kill bacteria, phagocytic ability, and macrophage-specific cytokine and chemokine production [5, 6]. Altered expression and function in the context of TLR are linked to the aging process. Some studies demonstrated a reduced TLR expression together with changes in cytokine release and macrophage polarization [22]. TLR stimulation, mediated by LPS (lipopolysaccharides), is usually responsible for IL-6 and TNF-*α* secretion, but in macrophages from old mice, a reduction in IL-6 and TNF-*α* and an increased production of IL-10 [22, 24] were observed. The aging effect on macrophages is the reduced expression of TLR1, TLR2, and TLR4, with reduction in proinflammatory cytokine production [25, 26]. Moreover, the overexpression of TLR3 may be involved in the establishment of viral infections in elderly individuals [12]. Actually, studies investigating the impact of aging on human monocyte cytokine production did not provide concordant results; indeed increased, unchanged, or decreased LPS-induced cytokine secretion has been reported [27, 28].

### 2.3. Microglia

Microglia are the resident immune cells of the CNS. They have the ability to detect molecules of injured CNS cells or invade pathogen infiltration by pattern recognition receptors expressed on their surface and on the surface of infiltrating monocytes. During aging, senescent microglia display a higher production of proinflammatory cytokines and proliferative capacity, and a reduction of chemotaxis and phagocytosis of Α*β* (amyloid-*β*) fibrils [29]. The number and density of microglial cells were higher in several aged brain areas, maybe to maintain the overall function. Replication of microglial cells can be very low in steady-state conditions and can be reactivated after perturbation by harmful stimulation [30], which can culminate in the shortening of telomeres and the realization of replicative senescence. In aged microglia, accumulation of mtDNA (mitochondrial DNA) damages leads to ROS overproduction [31] and accelerates the switch in the senescent microglial phenotypes. Aged microglia show morphological changes such as cytoplasmic hypertrophy, fragmentation, and loss of ramifications [32]. Several mechanisms are responsible for microglial aging phenotype, such as the loss of inhibitory ligand-receptor interactions [33], accumulation of misfolded proteins [34], and the chronic exposure to TGF- (transforming growth factor-) *β* that reduces the capacity of microglia to secrete anti-inflammatory cytokines. Expression of TLR1, TLR2, TLR4, TLR5, TLR7, and CD14 is upregulated in microglial cells with increasing age [35], while in the signaling of CX3CL1 (chemokine (C-X3-C motif) ligand 1)-CX3CR1 (receptor 1), CD (cluster of differentiation) 200-CD200R, and CD200, CX3CR1 is decreased in aged microglia, driving activation and extension of proinflammatory responses [36, 37].

### 2.4. Dendritic Cells

Dendritic cells represent an important bridge between innate and adaptive immune response. The plasmacytoid and the myeloid DC are antigen-presenting cells that detect pathogens through the expression of PRRs (pattern recognition receptors) [38], composed of TLR, RLRs (RIG-I-like receptors), NLR (Nod-like receptors), and ALRs (AIM2-like receptors) [39]. TLRs are the most investigated receptors in the aged condition, and their expression changes at extracellular and intracellular levels [12]. On the other hand, Agrawal et al., in 2007, showed no altered expression of TLR in monocyte-derived DCs, from aged and young humans [40], in accord with Guo et al., who confirmed age-related changes in aged C57BL/6 mice [41]. Moreover, in aged people, the alterations of DC functionality may affect the immune regulation. Zacca et al. investigated DC's ability to prime and activate naïve CD8+ T cells, showing a lower capacity, with negative impact on immune response, in aged people [42], against viral and bacterial infections. The numerical reduction of DC and the decreased IL-12 production cause the higher susceptibility to immunosenescence of the adaptive immune system [43].

### 2.5. Natural Killer

NK cells are defined as the innate cytotoxic lymphocytes [44], responsible for the early defense against pathogens and cancerous cells [12]. NK cells can be classified into two groups based on their CD56 surface expression. CD56^bright^ cells are the immature subset that showed a high proliferative activity and ability to release IFN*γ*, TNF-*α*, IL-10, RANTES, and MIP-1*α*, while CD56^dim^ cells, the mature subset, showed a high cytotoxic activity and lower ability to produce cytokines [7]. During the aging process, the composition of NK subsets may experience some alterations in number and function, and impairments of cytotoxicity and secretion of immune-regulatory cytokines and chemokines, a phenomenon referred to as NK cell immunosenescence. Many studies have demonstrated the presence of an increased number of total NK in old subjects, with raised CD56^dim^ [45–47], and lowered CD56^bright^ NK cells [45–48], with respect to young subjects. This change reflects the dysregulation of innate and adaptive immunity interaction, with reduction in chemokine production and in cytokine-induced proliferation [11]. IL-2-induced NK cell proliferation is decreased in old subjects [49], while induction of cytotoxicity by IL-2, IL-12, or IFN is maintained [50]. NK receptor expression and activation seem to be involved in the aging process, and several studies have shown an age-related decline in the percentage of NK cells expressing NKp30 or NKp46 [45, 46], while others have reported no age-dependent effect on the proportions of NK cells bearing these receptors [48]. NKG2D, CD16, and KIR expressions have been shown to be either maintained or increased with age, while a reduction of KLRG-1 and NKG2A is age-associated [47, 51]. Recent studies have shown that the presence of senescent cells may be related to a reduced clearance activity of NK cells [52].

## 3. Immunosenescence of Adaptive Immune Response Cells

The adaptive immune system is more recent, in evolutionary terms, than the innate immune system. It is able to adapt to new threats developing specific strategies against every challenge. It fails when the cells responsible for maintaining immune memory overcome the cells capable of taking action. Profound age-related changes occur in the adaptive immune system, contributing to decreased immune protection against infections and responses to vaccination. Changes in cells of the adaptive immune system appear to have an important impact on the ability to respond to the immune challenges [53].

### 3.1. B Cells

B cells follow a well-defined developmental process, starting from naïve cells, that does not produce a specific antibody isotype, to the establishment of the mature peripheral B cell pool, kept by self-renewal [10, 54]. B cell immunosenescence induces alterations starting from the generation during haematopoiesis to the reduction of cell diversity [55] and lower antibody specificity. Antibody specificity, affinity, and isotype switch are affected by aging, determining the increased susceptibility of the elderly to infectious diseases and reducing the protective effects of vaccination. As demonstrated by Frasca et al., there is a great impact on B cell surface Ig switch, in old than in young people [56]. During the aging process, in mouse models and in humans, the B cell switch in IgM to IgG, IgE, or IgA is decreased [57]. A possible mechanism could be the presence of defects in the *E2A*-encoded transcription factor E47, responsible for defining the antibody diversity and downregulating AID (activation-induced cytidine deaminase) and CSR (class switch recombination) in the B cells of aged people [58].

Loss of Ig diversity has been related to the reduced percentages and numbers of mature B cells during aging [58]. Some studies showed an increased expression of the activation markers CD27 and CD38 in mature B cell compartments [5, 58, 59]. This is confirmed by the lower levels of IgM and IgD in the elderly, underlining a shift from the naïve (CD27−) B cell subset towards the memory (CD27+) compartment [60]. B cell population is substantially altered in old age, contributing significantly to immunosenescence. The most important impairments affecting B cells during aging are reduction of the naïve B cell number, impaired capacity for response to new antigens, reduction of clonal expansion capability of memory cells related to reduction in circulating antibodies levels, and weakened antibodies function such as lower affinities and opsonizing abilities.

### 3.2. T Cells

Among the regulatory cells of the adaptive immunity, T cells are largely investigated in relation to immunosenescence. T cells are developed in the thymus, and distinct subsets are well recognized including the CD4+, CD8+, *γδ*, and NKT and the nonconventional T cells. All subsets have a specific role in the immune system [10, 61]. The thymus gland undergoes deterioration with aging, which starts after puberty and stabilizes after 65 years [10]. Thymus involution and reduced functionality are responsible for the reduction of naïve T cell frequency and number and for the increase in terminally differentiated T lymphocytes, with reduction in TCR (T-cell receptor) expression [62]. The numerical reduction in naïve T cells and TCR-reduced repertoire cause a decline in their functionality. In vitro studies showed that CD4+ naïve T cells, derived from old human and mice [63], have a reduced proliferation activity, an altered cytokine profile secretion, and a reduced responsiveness to TCR stimulation [63]. Otherwise, an increased number of memory CD4+ T cells is related to aging. Cytokine homeostasis results in alteration, favoring proinflammatory condition in aging. IL-6 increases in aged people and is responsible for inducing Th17 stimulation and related proinflammatory cytokine production [7, 64]. The CD8+ T cell subset is most affected by aging [65] with their accumulation. In particular T cell immunosenescence is characterized by increased number of highly differentiated memory CD8+, after chronic stimulation by viruses, like CMV (cytomegalovirus) infection [63]. As reported by Tu and Rao, CD8+ cells are able to persist after CMV infection, to prevent a virus reactivation. CD8+ subset presence during the time negatively impacts the immune system also in healthy CMV-infected individuals [66]. These alterations result in the impaired cellular immune response in infections and vaccinations [67].

**Figure 1:**
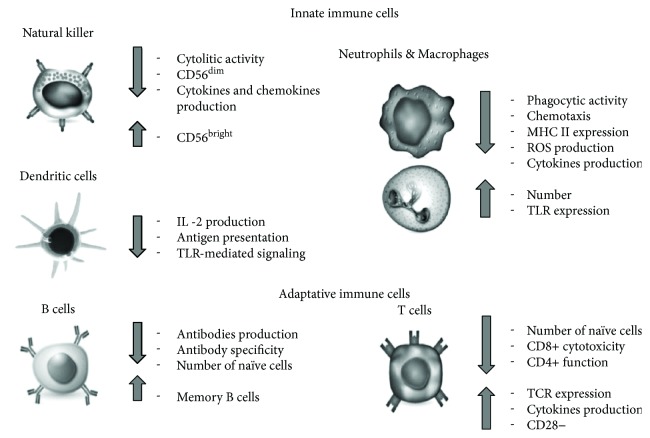
Schematic representation of immune cell age-related changes, leading to inflamm-aging, neurodegeneration, and neuroinflammation.

In association with thymus involution, age-related changes of T lymphocytes include the reduced expression of costimulatory molecules (CD28, CD27, CD40L), and a progressive accumulation of CD28 highly differentiated T cells. CD28+ cells mediate the TCR-induced proliferation and differentiation of naïve T cells. In CD28 gene knockout mouse, the involvement of CD28− T lymphocytes in age-dependent immune decline was demonstrated. CD28− cells are accumulated during life, due to their resistance to apoptosis. The loss of CD28 in CD4+ and CD8+ cells is followed by altered secretion of second messengers and altered signal pathway activation [64, 68] and lowering in immune response to vaccination in older people. Thus, CD28 loss in T cells can be defined as one of the aging hallmarks (Figure 1).

## 4. Immunosenescence and Inflamm-aging in Neurodegenerative Diseases

**Figure 2:**
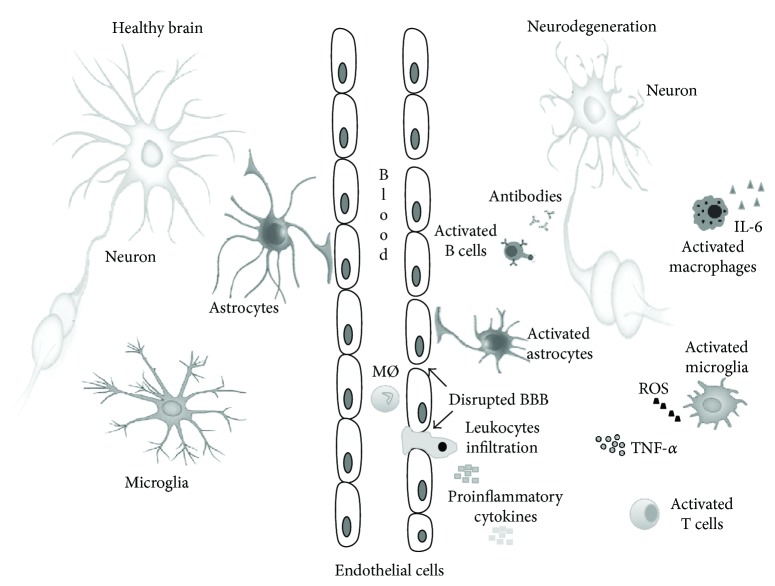
Immune activation in neurodegenerative diseases and healthy brain. The neurodegeneration is accompanied by immunosenescence and inflamm-aging. During aging, neuronal cells modify their morphology, and their overactivation leads to IL-6 and TNF-*α* abnormal production. The immune cells penetrate the damaged BBB and causes a further increase in proinflammatory cytokines, modulating, in turn, neuronal dysfunction.

The interaction between the nervous and immune systems during aging is characterized by bidirectional dependency and reciprocal causality of alterations. In elderly people, the increased systemic inflammatory condition, the inflamm-aging, and the peripheral immunosenescence can modulate neuronal immune cell activity and reactivity, leading to a chronic low-grade inflammation in the CNS, called neuro-inflamm-aging. Activation of glia by cytokines and glia proinflammatory productions are significantly involved in memory injury, and also in acute systemic inflammation, characterized by high levels of TNF-*α* and increase in the cognitive decline [69]. Autoreactive T cells, derived from the atrophied thymus, are a source of proinflammatory factors that strongly contribute to neurodegeneration. Immunosenescence and inflamm-aging induce brain aging, cognitive deficit, and memory loss; in fact, a bidirectional interconnection has been observed in neurodegenerative disorders, such as Alzheimer's disease (AD) and Parkinson's disease (PD) (Figure 2).

### 4.1. Alzheimer's Disease

Alzheimer's disease is the most common type of age-related neuronal disorder, affecting 44 million people in the world [70, 71]; with the improvement of life expectancy, this number is constantly growing. People that may develop AD in the late life, at the age of 65 and older [72], present deficit in memory, language, spatial vision, and physical equilibrium that together lead to cognitive impairment [70, 72].

Characteristics of AD are amyloid-beta (A*β*) deposition, NFTs (neurofibrillary tangles), NP (neuritic plaques), and the activation of immune cells of the CNS, microglia, and astrocytes [73]. A*β* deposition is the central event in AD pathogenesis and derived from larger and hyperactivation of the APP (amyloid precursor protein) [53]. APP is a transmembrane glycoprotein responsible for binding proteoglycans for regulating intracellular processes such as neuron-cell and cell-matrix interaction, cell growth, and synaptic plasticity [74]. The pathophysiological relation between A*β* and tau protein is still unclear [53, 70]. NFTs are composed of hyperphosphorylated tau protein, responsible for stabilizing microtubules in neurons, and their formation is secondary to neuronal damage and A*β* deposition. In regions of A*β* deposits and NFT, signs of oxidative stress and high levels of inflammatory mediators were observed. The inflammatory response is necessary and crucial to combat pathogen or dying cell, but dysregulated inflammatory responses are responsible for tissue damage such as in the CNS inflammation. To the inflammatory response in the CNS, cells of immune system, cells of the CNS, adhesion molecules, cytokines, and chemokines take part. Numerous studies have assessed the association between immune system activation and AD [71, 75]. This relation can be explained by the highly regulated communication between the brain and the immune system [76], and Sutherland et al., in 2015, explained the role of the immune cells in relation to the inflammatory condition inside and outside the CNS [77]. A*β* deposition-induced AD pathogenesis brings the activation of microglial and astrocytes cells, the phagocytosis and degradation of *β*-amyloid, and elimination of the debris of dead or dying cells, thereby reducing the likelihood of further cell loss through the release of toxic agents. Furthermore, reactive astrocytes isolate neurons from senile plaques and release cytokines and growth factors that may help damaged neurons to survive and promote repair to inhibit Alzheimer disease [71]. The natural decline of CNS immune cells in adult and elderly people leads to a reduced state of health of the brain, contributing to AD. Microglial and astrocytes cell uncontrolled activation, increased in an age-dependent manner, leads to excessive inflammation [78]. Inflammation can lead to the injury or death of neurons, particularly if the response is chronic and uncontrolled. Neuronal loss in AD may be a direct effect, or due to the secretion of neurotoxins by activated microglia. Activated microglia may be involved in the generation of senile plaque either by the secretion of A*β*1–42 or through the release of agents such as iron, which aggregates soluble *β*-amyloid fragments. Microglia and astrocytes produced proinflammatory component, and also neurons, oligodendrocytes, and vascular endothelial cells may contribute to the maintenance of the inflammatory environment. Activated microglia may promote neurodegeneration but could also play a neuroprotective role dependently by the context, timing, and mediator of the inflammatory response. Cytokines are multifunctional mediators that act in a context-dependent manner and can promote or inhibit inflammatory processes. Thus, TNF-*α* may promote an inflammatory response, but it may protect neurons or even modulate neurotransmission, or TGF-*β*1 may promote inflammation and cellular infiltration early in an immune response, but it is critical later in downregulating inflammation [79]. In Dr. Chakrabarty's laboratory, using transgenic mice as AD model, it was highlighted that mIL-6-mediated reactive gliosis may be helpful early in the disease development by possibly improving A*β* plaque clearance rather than facilitating a neurotoxic feedback loop that aggravates A*β* plaque pathology [80], likely by inducing microglial phagocytosis and shifting towards the alternative M2 microglia with an anti-inflammatory phenotype.

The bidirectional communication between the nervous and immune systems, when properly orchestrated, resulted in body protection, and cytokines might be the key molecules that initiate the immune-to-brain communication, and activation of specific cytokine-to-brain pathways differentially mediates response to specific events. Peripherally released cytokines may reach the brain through permeable regions of the blood-brain barrier (BBB), by activation of nonneuronal cells in the BBB that can initiate a cascade of neural communication events, by active transporters that allow cytokines to cross the BBB, or by vagus nerve that acts as a neural route.

The chronicity of inflammation is characterized by activation of monocytes, as well as macrophages and infiltration in the CNS [81]. Peripheral immune system cell overactivation determines the increased proinflammatory cytokine and chemokine release, with upregulation of immune receptor expression (MHC II, CD68, CD14, CD11, and TLRs) [71, 81], promoting the brain tissue damage. Activated immune cells and their products reach the CNS crossing the BBB, physiologically responsible for isolating the brain from peripheral circulation. Lee and collaborators have shown an increased BBB permeability in aged mice, with a reduced expression of TJs (tight junctions) inhibiting endothelial cell interconnection [82]. In recent studies on 12-month-old wild-type C57BL/6J mice, BBB dysfunction was demonstrated in relation to TJs lost and heightened proinflammatory cytokine expression, in particular TNF-*α* [83], IFN*γ* [41], IL-1*β*, IL-6, and IL-18 [84]. These cytokines are produced not only by overactivated neuronal immune cells but also by peripheral immune cells, showing a relationship with neuronal dysfunction, increased inflammation in the brain parenchyma, and cognitive decline [85–87].

During the AD pathogenesis, the brain damage and the BBB higher permeability define a selective entry of peripheral immune cells in the CNS that activate the immune response [88], as the T cell infiltration in brain tissue reported by McGeer et al. [89], in a *postmortem* brain analysis [90].

In vitro studies demonstrate that A*β* stimulation of microglial cells and astrocytes is responsible for increasing the levels of TNF-*α* and TGF-*β*1 production [91], which are known to promote T cell transmigration [88]. The increased expression of MHC I and II represents the mechanism by which T cell numbers increase in the brain [53]. Moreover, there is a higher expression of CCR5 on B cell surface in the presence of A*β* that leads to inflammation and cytokine and chemokine production [92]. Marsh et al., in 2016, investigated the mechanism by which B cells are involved in AD progression using the immune-deficient transgenic model of AD, Rag-5xf AD mice, showing that A*β* plaque deposition is favored by nonamyloid-reactive IgG [93].

In mouse models, it has been observed that neurotoxicity could be mediated by activation of NLRP3 (NACHT, LRR, and PYD domains-containing protein 3), with release of classical proinflammatory molecules, such as IL-1*β*, IL-18, and IL-1*α* [94], and activation of macrophages. Monocytes/macrophages represent, not in pathological conditions, the responder cells to inflammatory stimuli and stressors by expressing protective molecules, such as IL-12 and IL-23, which contribute to homeostasis regulation, also in the perivascular space, through phagocytic activity [95]. Monocytes infiltrate the brain in AD, crossing the BBB and assuming macrophages or dendritic cell phenotype, with different states of activation [75]. In an A*β*-induced inflammatory microenvironment, macrophages change their protective phenotype M2 [96] to the proinflammatory M1, involved in the production of IL-1*β* and TNF-*α* and phagocytosis of dangerous molecules produced by stressors [75, 97]. These cells interact with microglia and A*β* plaques, considered as an inflammasome activator, producing cytokines and ROS, leading to neuronal loss and apoptosis [88]. To assess the immune system involvement, many authors evaluated the circulating levels of IL-6, TNF-*α*, and IFN*γ* production in AD patient serum/plasma [53, 98, 99], CSF (cerebrospinal fluid) [100], and derived peripheral blood mononuclear cells [101]. Circulating levels of proinflammatory cytokines are elevated and significantly associated with increased risk for AD cognitive decline. This increase was found also in triple-transgenic mice models of AD (3xTg-AD mice), considered as the most similar to human AD model [102]. Most recently in 3xTg-AD mice, an increased proinflammatory response with IL-6 and TNF-*α* increased production has been observed, in association with immune cell infiltration in the brain [103].

### 4.2. Parkinson's Disease

Parkinson's disease is a neurodegenerative age-related disorder [104] affecting 1% of 60-aged human population and is considered the second most common neurodegenerative disease [105]. PD patient's clinical signs are bradykinesia, rigidity, and tremor, usually manifesting unilaterally or at least asymmetrically, in addition to sensory and neuropsychiatric features [106, 107].

The pathogenesis of PD is related to people's exposure to many environmental risk factors, such as pesticides, heavy metal, welding and chemical agent exposure [104, 108], and genetic and epigenetic factors [105], leading to oxidative stress, proteasomal system dysfunction, protein aggregation, and misfolding. These alterations are common to changes that occur during aging [108]. The effects of aging refer to the physiological changes of neuronal and nonneuronal cells [104], including progressive degeneration and loss of DA neurons in the midbrain substantia nigra, reduction of nigral pigmented neurons, accumulation of alpha-synuclein, and the inhibition of the UPS (ubiquitin proteasome system) [109]. The accumulation of *α*-synuclein leads to lamellated eosinophilic cytoplasmic inclusions, called “Lewy bodies” in the neuronal body, and to the insoluble polymers (Lewy neurites) in neuronal processes, astrocytes, and oligodendroglial cells [104, 67].

In the postmortem PD patients' substantia nigra, the content of DA (dopamine) is reduced by 10% compared to normal values, associated with dopaminergic neurons loss of cellular bodies [104].

It has been demonstrated that neurodegeneration and immune system activation are increased with age and contribute to PD onset [105, 108].

The common features of immune cell involvement are represented by the establishment of inflammation in the brain, recruitment of peripheral immune cells, proinflammatory mediators production, and increased ROS concentration. All of these may increase the neurodegenerative process in aged people [105], with acceleration and increased prevalence of PD [108].

Peripheral immune system activation affects brain neuroinflammation, for example, exacerbating the microglial function [110] and defining changes in DA neurons. In aged brain, microglial cells increase the MHC II and TLR expressions and adopt a proinflammatory phenotype to stimulate the peripheral immune cell migration in the CNS [106]. In addition, astrocytes show an increased proinflammatory profile with higher MCP-1 secretion for priming peripheral monocytes [111]. Peripheral immune cells, like macrophages and monocytes, in healthy brain, are responsible for the immuno-surveillance and the production of proinflammatory cytokines, such as TNF-*α* and IL-6, to solve the injury. The immunosenescence of these cells, with reduction of phagocytic activity and proinflammatory cytokine and chemokine production, seems to influence the disease progression because of the lack of surveillance [67]. Most recently, Lindenau et al. showed the involvement of TNF-*α*, which appears to be significantly released by monocytes/macrophage with proinflammatory phenotype. In elderly individuals, immunosenescent cells seems to contribute to the increased expression of TNF-*α*. The hypothesized mechanism is the methylation of the gene responsible for TNF production, which can contribute to the progression of inflammation and to the aging process [112].

Together, these data support the hypothesis that altered innate immune system activation, such as macrophages and monocytes, directly contributes to the pathology and biology of PD.

In PD patients, the most relevant sign is the alteration of the lymphocyte subsets. A reduction in the total number has been observed for CD19+ B cells and CD3+ T cells. Among T cells, their activation is related to the DC infiltration in lymph nodes and cell stimulation [105]. In PD patients, in association with immunosenescence, the reduced count and functionality of DC are responsible for the reduction in T cell activation [113]. The BBB dysfunction in PD patients determines the CD4+ and CD8+ infiltration in the CNS [106, 114]. Circulating levels of CD4+ T cells decrease in PD, while CD8+ T cells are unchanged, promoting the immune aging [115]. Baba et al. have demonstrated the selective reduction of CD4+CD45RA+ phenotype, naïve cells and an increase or a nonalteration in the expression of CD4+CD45RA− memory cells. This is supported by studies in animal models and in humans, highlighting the involvement of Treg (regulatory T cell) lymphocytes in the promotion of immune-mediated diseases in aged people [116]. In animal models, such as 6-hydroxydopamine (6-OHDA) PD rats, the increased expression of CD4+ T cells is responsible for increasing the inflammatory cytokine expression and facilitates NM (neuromelanin) activation and B cell production of autoantibody [117]. Additionally, the progressively increased neuroinflammation drives a high cognitive decline linked to vulnerability to virus and bacterial infection in aged people [67]. The reduced diversity in the T cell repertoire also represents one of the causal factors of the deregulation of the immune response in the elderly, with a significant increase of cytokine levels. Several studies, in which CSF and serum of PD patients were analyzed and correlated to PD progression, support this hypothesis. Increased levels of TNF-*α*, IL-1*β*, IL-2, IL-4, and IL-6 were detected in CSF of PD patients compared to age-matched controls [117]. Additionally, the increased levels of inflammatory markers were detected in serum from PD patients [105, 118, 119]. Moreover, circulating levels of cytokines were correlated to the overactivation of T and B lymphocytes, and upregulation of microglial cells, which are able to induce in turn IFN*γ* and TNF-*α* expressions [113, 115, 120]. It is well established that pathological changes in the CNS can be evaluated the in periphery. New proposed targets are circulating microvesicles that are generated in response to intracellular stimuli, representing an interconnection between normal and pathological tissues. Peripheral systems analysis of PD patients has led to highlighting the presence of brain damage, and microvesicle biology can be important for pathogenesis uptake.

Many authors evaluate the *α*-synuclein secretion outside or within exosomes [121, 122]. All of these studies support the hypothesis that *α*-synuclein associated with exosomes contributes to the progression of brain disorders, but the mechanism is currently unclear. In addition, it was demonstrated that the exosome release in pathogenic form is upregulated in association with PD mutated genes, like LRRK2 and ATP13A2, that acts in modulating microvesicle biogenesis and trafficking.

## 5. Conclusions

Over the decades, there is enormous progress in the neural-immune crosstalk and mutual regulation of aging and age-related diseases, and in describing the innate and adaptive immune age-related alterations; however, investigations are necessary. Several studies have shown that changes in cell number, activity, and receptor expression lead, as consequence, to an increased susceptibility to infectious and inflammatory age-related diseases, in elderly population. Evidences showed the association between immunosenescence with a low-grade chronic inflammation, called the inflamm-aging, although inflamm-aging is necessary but not sufficient to cause age-related neurodegenerative diseases. The increased proinflammatory environment could be the major contributing factor to the development of aging-associated diseases. Given the well-established communication between the immune system and brain, the age-related immune dysregulation may bring neurodegeneration. Several studies have demonstrated that immunosenescence and inflamm-aging can induce an overactivation of CNS immune cells, promoting neuroinflammation. In AD patients, the microglial aging and dysfunction lead to A*β* accumulation and loss of peripheral immune response, contributing to disease pathogenesis. Furthermore, in PD, the interaction between aging and over time decreased immune response suggests a disease predisposition for neurodegeneration. Recently, several studies have reported the relationship between delayed immunological aging and reduced expansion of senescent late-stage differentiated T cells and active lifestyle and has been suggested that aerobic exercise training might attenuate cognitive impairment and reduce dementia risk. Although it is unknown whether effects of exercise are direct, such as a targeted removal of dysfunctional T cells, or indirect, such as lower inflammatory activity, it may be hypothesized that these changes can provide benefits for the health, including mitigate cognitive impairment. To mitigate the decline in the immune function, a practical and economic approach is represented by the nutritional intervention, without forgetting that difference exists between nutritional interventions and their immune-modulating activity. The use of both probiotics and prebiotics may reduce immunosenescence, improving Treg homeostasis, reducing the colonization potential of pathogens, and counteracting chronic inflammation, and may positively affect cognitive function [123, 124].

Caloric restriction partially retards or restores age-associated immunosenescence by oxidative stress energy metabolism regulation, and reduction of proinflammatory cytokine production and neuroendocrine homeostasis [125]. A healthy lifestyle may help to retard immunosenescence; in fact good sleep duration improves immune functions, and poor sleep may affect the body's ability to clear the amyloid-beta from the brain, while stress reduces the effectiveness of the immune system and can cause damage to the brain.

New strategies to combat immunosenescence and neurodegeneration are focused on cellular and genetic therapies, such as genetic reprogramming and bone marrow transplantation, but cell reprogramming has still poor efficiency, and clinical translation shows several ethical and safety questions that may be answered.

**Figure 3:**
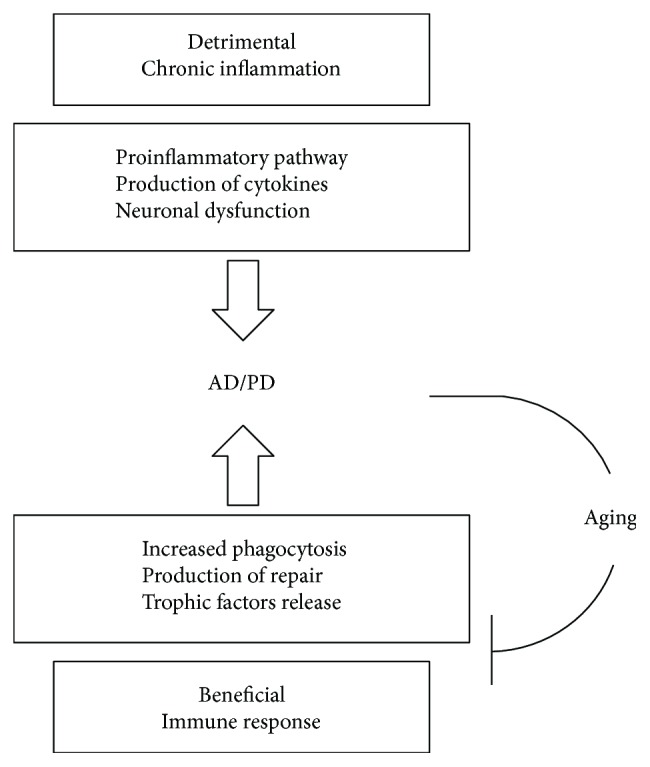
Advantages and disadvantages of an inflammatory response. Phagocytosis eliminates the debris of dead or dying cells, thereby reducing the likelihood of further cell loss through the release of toxic agents. Cytokines and growth factors may help damaged neurons to survive and promote repair. Chronic inflammation can lead to the injury or death of neurons.

Thus, a better understanding of immunosenescence mechanisms will be necessary to develop new, unconventional, or pharmacological therapy strategies, for peripheral and CNS immunosenescence delay. Additional studies are required to determine the effectiveness and optimal conditions to improve the function of the aged immune system and undertake the challenges of immunosenescence. Immunosenescence reversion can prevent, in elderly individuals, chronic inflammation and associated neurodegenerative diseases and can provide new and additional target for improving healthy lifespan and slow down age-related diseases incidence (Figure 3).

## References

[1] Balistreri C. R., Candore G., Accardi G., Colonna-Romano G., Lio D. (2013). NF-*κ*B pathway activators as potential ageing biomarkers: targets for new therapeutic strategies. *Immunity & Ageing*.

[2] Khan S. S., Singer B. D., Vaughan D. E. (2017). Molecular and physiological manifestations and measurement of aging in humans. *Aging Cell*.

[3] Chaplin D. D. (2010). Overview of the immune response. *Journal of Allergy and Clinical Immunology*.

[4] Pawelec G. (2017). Immunosenescence and cancer. *Biogerontology*.

[5] Boren E., Gershwin M. E. (2004). Inflamm-aging: autoimmunity, and the immune-risk phenotype. *Autoimmunity Reviews*.

[6] Weiskopf D., Weinberger B., Grubeck-Loebenstein B. (2009). The aging of the immune system. *Transplant International*.

[7] Ponnappan S., Ponnappan U. (2011). Aging and immune function: molecular mechanisms to interventions. *Antioxidants & Redox Signaling*.

[8] Franceschi C., Bonafe M., Valensin S. (2000). Inflamm-aging. An evolutionary perspective on immunosenescence. *Annals of the New York Academy of Sciences*.

[9] Qin L., Jing X., Qiu Z. (2016). Aging of immune system: immune signature from peripheral blood lymphocyte subsets in 1068 healthy adults. *Aging*.

[10] Simon A. K., Hollander G. A., McMichael A. (2015). Evolution of the immune system in humans from infancy to old age. *Proceedings of the Royal Society B: Biological Sciences*.

[11] Solana R., Tarazona R., Gayoso I., Lesur O., Dupuis G., Fulop T. (2012). Innate immunosenescence: effect of aging on cells and receptors of the innate immune system in humans. *Seminars in Immunology*.

[12] Shaw A. C., Joshi S., Greenwood H., Panda A., Lord J. M. (2010). Aging of the innate immune system. *Current Opinion in Immunology*.

[13] Lord J. M., Butcher S., Killampali V., Lascelles D., Salmon M. (2001). Neutrophil ageing and immunesenescence. *Mechanisms of Ageing and Development*.

[14] Verschoor C. P., Loukov D., Naidoo A. (2015). Circulating TNF and mitochondrial DNA are major determinants of neutrophil phenotype in the advanced-age, frail elderly. *Molecular Immunology*.

[15] Minet-Quinard R., Farges M. C., Thivat E. (2010). Neutrophils are immune cells preferentially targeted by retinoic acid in elderly subjects. *Immunity & Ageing*.

[16] Sauce D., Dong Y., Campillo-Gimenez L. (2016). Reduced oxidative burst by primed neutrophils in the elderly individuals is associated with increased levels of the CD16^bright^/CD62L^dim^ immunosuppressive subset. *The Journals of Gerontology Series A: Biological Sciences and Medical Sciences*.

[17] Bartlett D. B., Shepherd S. O., Wilson O. J. (2017). Neutrophil and monocyte bactericidal responses to 10 weeks of low-volume high-intensity interval or moderate-intensity continuous training in sedentary adults. *Oxidative Medicine and Cellular Longevity*.

[18] Geissmann F., Manz M. G., Jung S., Sieweke M. H., Merad M., Ley K. (2010). Development of monocytes, macrophages, and dendritic cells. *Science*.

[19] Linton P., Thoman M. (2014). Immunosenescence in monocytes, macrophages, and dendritic cells: lessons learned from the lung and heart. *Immunology Letters*.

[20] De La Fuente M. (1985). Changes in the macrophage function with aging. *Comparative Biochemistry and Physiology Part A: Physiology*.

[21] Hearps A. C., Martin G. E., Angelovich T. A. (2012). Aging is associated with chronic innate immune activation and dysregulation of monocyte phenotype and function. *Aging Cell*.

[22] Linehan E., Fitzgerald D. C. (2015). Ageing and the immune system: focus on macrophages. *European Journal of Microbiology and Immunology*.

[23] Herrero C., Marques L., Lloberas J., Celada A. (2001). IFN-gamma dependent transcription of MHC class II IA is impaired in macrophages from aged mice. *The Journal of Clinical Investigation*.

[24] Boehmer E. D., Goral J., Faunce D. E., Kovacs E. J. (2004). Age-dependent decrease in toll-like receptor 4-mediated proinflammatory cytokine production and mitogen-activated protein kinase expression. *Journal of Leukocyte Biology*.

[25] Qian F., Montgomery R. R. (2012). Quantitative imaging of lineage-specific toll-like receptor-mediated signaling in monocytes and dendritic cells from small samples of human blood. *Journal of Visualized Experiments*.

[26] Pantsulaia I., Ciszewski W. M., Niewiarowska J. (2016). Senescent endothelial cells: potential modulators of immunosenescence and ageing. *Ageing Research Reviews*.

[27] O’Mahony L., Holland J., Jackson J., Feighery C., Hennessy T. P. J., Mealy K. (1998). Quantitative intracellular cytokine measurement: age-related changes in proinflammatory cytokine production. *Clinical and Experimental Immunology*.

[28] Beharka A. A., Meydani M., Wu D., Leka L. S., Meydani A., Meydani S. N. (2001). Interleukin-6 production does not increase with age. *The Journals of Gerontology Series A: Biological Sciences and Medical Sciences*.

[29] Floden A. M., Combs C. K. (2011). Microglia demonstrate age-dependent interaction with amyloid-*β* fibrils. *Journal of Alzheimer’s Disease*.

[30] Wong W. T. (2013). Microglial aging in the healthy CNS: phenotypes, drivers, and rejuvenation. *Frontiers in Cellular Neuroscience*.

[31] Corral-Debrinski M., Horton T., Lott M. T., Shoffner J. M., Beal M. F., Wallace D. C. (1992). Mitochondrial DNA deletions in human brain: regional variability and increase with advanced age. *Nature Genetics*.

[32] Damani M. R., Zhao L., Fontainhas A. M., Amaral J., Fariss R. N., Wong W. T. (2011). Age-related alterations in the dynamic behavior of microglia. *Aging Cell*.

[33] Perry V. H., Holmes C. (2014). Microglial priming in neurodegenerative disease. *Nature Reviews Neurology*.

[34] Flanary B. E., Sammons N. W., Nguyen C., Walker D., Streit W. J. (2007). Evidence that aging and amyloid promote microglial cell senescence. *Rejuvenation Research*.

[35] Letiembre M., Hao W., Liu Y. (2007). Innate immune receptor expression in normal brain aging. *Neuroscience*.

[36] Bachstetter A. D., Morganti J. M., Jernberg J. (2011). Fractalkine and CX 3 CR1 regulate hippocampal neurogenesis in adult and aged rats. *Neurobiology of Aging*.

[37] Wynne A. M., Henry C. J., Huang Y., Cleland A., Godbout J. P. (2010). Protracted downregulation of CX_3_CR1 on microglia of aged mice after lipopolysaccharide challenge. *Brain, Behavior, and Immunity*.

[38] Agrawal A., Gupta S. (2011). Impact of aging on dendritic cell functions in humans. *Ageing Research Reviews*.

[39] Hayashi T., Nakamura T., Takaoka A. (2011). Pattern recognition receptors. *Japanese Journal of Clinical Immunology*.

[40] Agrawal A., Agrawal S., Cao J. N., Su H., Osann K., Gupta S. (2007). Altered innate immune functioning of dendritic cells in elderly humans: a role of phosphoinositide 3-kinase-signaling pathway. *Journal of Immunology*.

[41] Guo Z., Tilburgs T., Wong B., Strominger J. L. (2014). Dysfunction of dendritic cells in aged C57BL/6 mice leads to failure of natural killer cell activation and of tumor eradication. *Proceedings of the National Academy of Sciences of the United States of America*.

[42] Zacca E. R., Crespo M. I., Acland R. P. (2015). Aging impairs the ability of conventional dendritic cells to cross-prime CD8^+^ T cells upon stimulation with a TLR7 Ligand. *PloS One*.

[43] Della Bella S., Bierti L., Presicce P. (2007). Peripheral blood dendritic cells and monocytes are differently regulated in the elderly. *Clinical Immunology*.

[44] Panda A., Arjona A., Sapey E. (2009). Human innate Immunosenescence: causes and consequences for immunity in old age. *Trends in Immunology*.

[45] Hazeldine J., Lord J. M. (2013). The impact of ageing on natural killer cell function and potential consequences for health in older adults. *Ageing Research Reviews*.

[46] Almeida-Oliveira A., Smith-Carvalho M., Porto L. C. (2011). Age-related changes in natural killer cell receptors from childhood through old age. *Human Immunology*.

[47] Lutz C. T., Karapetyan A., Al-Attar A. (2011). Human NK cells proliferate and die in vivo more rapidly than T cells in healthy young and elderly adults. *Journal of Immunology*.

[48] Le Garff-Tavernier M., Béziat V., Decocq J. (2010). Human NK cells display major phenotypic and functional changes over the life span. *Aging Cell*.

[49] Takahashi Y., Kuro-o M., Ishikawa F. (2000). Aging mechanisms. *Proceedings of the National Academy of Sciences of the United States of America*.

[50] Gayoso I., Sanchez-Correa B., Campos C. (2011). Immunosenescence of human natural killer cells. *Journal of Innate Immunity*.

[51] Hayhoe R. P., Henson S. M., Akbar A. N., Palmer D. B. (2010). Variation of human natural killer cell phenotypes with age: identification of a unique KLRG1-negative subset. *Human Immunology*.

[52] Sagiv A., Burton D. G., Moshayev Z. (2016). NKG2D ligands mediate immunosurveillance of senescent cells. *Aging*.

[53] Martorana A., Bulati M., Buffa S. (2012). Immunosenescence, inflammation and Alzheimer’s disease. *Longevity & Healthspan*.

[54] Miller J. P., Cancro M. P. (2007). B cells and aging: balancing the homeostatic equation. *Experimental Gerontology*.

[55] Buffa S., Bulati M., Pellicanò M. (2011). B cell immunosenescence: different features of naïve and memory B cells in elderly. *Biogerontology*.

[56] Frasca D., Van der Put E., Riley R. L., Blomberg B. B. (2004). Reduced Ig class switch in aged mice correlates with decreased E47 and activation-induced cytidine deaminase. *Journal of Immunology*.

[57] Bagnara D., Squillario M., Kipling D. (2015). A reassessment of IgM memory subsets in humans. *Journal of Immunology*.

[58] Frasca D., Landin A. M., Lechner S. C. (2008). Aging down-regulates the transcription factor E2A, activation-induced cytidine deaminase, and Ig class switch in human B cells. *Journal of Immunology*.

[59] Frasca D. (2017). Senescent B cells in aging and age-related diseases: their role in the regulation of antibody responses. *Experimental Gerontology*.

[60] Colonna-Romano G., Bulati M., Aquino A. (2009). A double-negative (IgD−CD27−) B cell population is increased in the peripheral blood of elderly people. *Mechanisms of Ageing and Development*.

[61] Jagger A., Shimojima Y., Goronzy J. J., Weyand C. M. (2014). Regulatory T cells and the immune aging process: a mini-review. *Gerontology*.

[62] Pinti M., Appay V., Campisi J. (2016). Aging of the immune system—focus on inflammation and vaccination. *European Journal of Immunology*.

[63] Weng N. (2006). Aging of the immune system: how much can the adaptive immune system adapt?. *Immunity*.

[64] Watad A., Bragazzi N. L., Adawi M. (2017). Autoimmunity in the elderly: insights from basic science and clinics—a mini-review. *Gerontology*.

[65] Coder B., Wang W., Wang L., Wu Z., Zhuge Q., Su D. M. (2017). Friend or foe: the dichotomous impact of T cells on neuro-de/re-generation during aging. *Oncotarget*.

[66] Tu W., Rao S. (2016). Mechanisms underlying T cell immunosenescence: aging and cytomegalovirus infection. *Frontiers in Microbiology*.

[67] Liang Z., Zhao Y., Ruan L. (2017). Impact of aging immune system on neurodegeneration and potential immunotherapies. *Progress in Neurobiology*.

[68] Vallejo A. N. (2005). CD28 extinction in human T cells: altered functions and the program of T-cell senescence. *Immunological Reviews*.

[69] Holmes C., Cunningham C., Zotova E. (2009). Systemic inflammation and disease progression in Alzheimer disease. *Neurology*.

[70] Lane C. A., Hardy J., Schott J. M. (2017). Alzheimer’s disease. *European Journal of Neurology*.

[71] Deleidi M., Jäggle M., Rubino G. (2015). Immune aging, dysmetabolism, and inflammation in neurological diseases. *Frontiers in Neuroscience*.

[72] Scheltens P., Blennow K., Breteler M. (2016). Alzheimer’s disease. *The Lancet*.

[73] Thal D. R., Rüb U., Orantes M., Braak H. (2002). Phases of A beta-deposition in the human brain and its relevance for the development of AD. *Neurology*.

[74] Müller U. C., Zheng H. (2012). Physiological functions of APP family proteins. *Cold Spring Harbor Perspectives in Medicine*.

[75] Di Benedetto S., Müller L., Wenger E., Düzel S., Pawelec G. (2017). Contribution of neuroinflammation and immunity to brain aging and the mitigating effects of physical and cognitive interventions. *Neuroscience & Biobehavioral Reviews*.

[76] Richartz E., Stransky E., Batra A. (2005). Decline of immune responsiveness: a pathogenetic factor in Alzheimer’s disease?. *Journal of Psychiatric Research*.

[77] Sutherland K., Li T., Cao C. (2015). Alzheimer’s disease and the immune system. *Symbiosis Online Journal*.

[78] Luo X. G., Ding J. Q., Chen S. D. (2010). Microglia in the aging brain: relevance to neurodegeneration. *Molecular Neurodegeneration*.

[79] Wyss-Coray T. (2006). Inflammation in Alzheimer disease: driving force, bystander or beneficial response?. *Nature Medicine*.

[80] Chakrabarty P., Jansen-West K., Beccard A. (2010). Massive gliosis induced by interleukin-6 suppresses A*β* deposition in vivo: evidence against inflammation as a driving force for amyloid deposition. *The FASEB Journal*.

[81] Rubio-Perez J. M., Morillas-Ruiz J. M. (2012). A review: inflammatory process in Alzheimer's disease, role of cytokines. *The Scientific World Journal*.

[82] Lee P., Kim J., Williams R. (2012). Effects of aging on blood brain barrier and matrix metalloproteases following controlled cortical impact in mice. *Experimental Neurology*.

[83] Elahy M., Jackaman C., Mamo J. C. L. (2015). Blood–brain barrier dysfunction developed during normal aging is associated with inflammation and loss of tight junctions but not with leukocyte recruitment. *Immunity & Ageing*.

[84] Dansokho C., Ait Ahmed D., Aid S. (2016). Regulatory T cells delay disease progression in Alzheimer-like pathology. *Brain*.

[85] Simen A. A., Bordner K. A., Martin M. P., Moy L. A., Barry L. C. (2011). Cognitive dysfunction with aging and the role of inflammation. *Therapeutic Advances in Chronic Disease*.

[86] Humpel C., Hochstrasser T. (2011). Cerebrospinal fluid and blood biomarkers in Alzheimer’s disease. *World Journal of Psychiatry*.

[87] Lim A., Krajina K., Marsland A. L. (2013). Peripheral inflammation and cognitive aging. *Modern Trends in Pharmacopsychiatry*.

[88] Hohsfield L. A., Humpel C. (2015). Migration of blood cells to *β*-amyloid plaques in Alzheimer's disease. *Experimental Gerontology*.

[89] McGeer P. L., Yasojima K., McGeer E. G. (2001). Inflammation in Alzheimer’s disease. *Archives of Neurology Journal*.

[90] Togo T., Akiyama H., Isekiet E. (2002). Occurrence of T cells in the brain of Alzheimer’s disease and other neurological diseases. *Journal of Neuroimmunology*.

[91] Mietelska-Porowska A., Wojda U. (2017). T lymphocytes and inflammatory mediators in the interplay between brain and blood in Alzheimer's disease: potential pools of new biomarkers. *Journal of Immunology Research*.

[92] Pellicanò M., Larbi A., Goldeck D. (2012). Immune profiling of Alzheimer patients. *Journal of Neuroimmunology*.

[93] Marsh S. E., Abud E. M., Lakatos A. (2016). The adaptive immune system restrains Alzheimer’s disease pathogenesis by modulating microglial function. *Proceedings of the National Academy of Sciences*.

[94] Sheedy F. J., Grebe A., Rayner K. J. (2013). CD36 coordinates NLRP3 inflammasome activation by facilitating intracellular nucleation of soluble ligands into particulate ligands in sterile inflammation. *Nature Immunology*.

[95] Murray P. J., Wynn T. A. (2011). Protective and pathogenic functions of macrophage subsets. *Nature Reviews Immunology*.

[96] Heneka M. T., Kummer M. P., Stutz A. (2013). NLRP3 is activated in Alzheimer’s disease and contributes to pathology in APP/PS1 mice. *Nature*.

[97] Thériault P., El Ali A., Rivest S. (2015). The dynamics of monocytes and microglia in Alzheimer’s disease. *Alzheimer's Research & Therapy*.

[98] Reale M., Iarlori C., Gambi F., Lucci I., Salvatore M., Gambi D. (2005). Acetylcholinesterase inhibitors effects on oncostatin-M, interleukin-1 beta and interleukin-6 release from lymphocytes of Alzheimer’s disease patients. *Experimental Gerontology*.

[99] Banerjee A., Khemka V. K., Roy D. (2017). Role of pro-inflammatory cytokines and vitamin D in probable Alzheimer's disease with depression. *Aging and Disease*.

[100] Wang W. Y., Tan M. S., Yu J. T., Tan L. (2015). Role of pro-inflammatory cytokines released from microglia in Alzheimer's disease. *Annals of Translational Medicine*.

[101] Speciale L., Calabrese E., Saresella M. (2008). Lymphocyte subset patterns and cytokine production in Alzheimer's disease patients. *Neurobiology of Aging*.

[102] Gimènez-Llort L., Matè I., Manassra R., Vida C., De la Fuente M. (2012). Peripheral immune system and neuroimmune communication impairment in a mouse model of Alzheimer’s disease. *Annals of the New York Academy of Sciences*.

[103] Montacute R., Foley K., Forman R., Else K. J., Cruickshank S. M., McRae Allan S. (2017). Enhanced susceptibility of triple transgenic Alzheimer’s disease (3xTg-AD) mice to acute infection. *Journal of Neuroinflammation*.

[104] Rodriguez M., Rodriguez-Sabate C., Morales I., Sanchez A., Sabate M. (2015). Parkinson's disease as a result of aging. *Aging Cell*.

[105] Calabrese V., Santoro A., Monti D. (2018). Aging and Parkinson's disease: inflammaging, neuroinflammation and biological remodeling as key factors in pathogenesis. *Free Radical Biology & Medicine*.

[106] Hindle J. V. (2010). Ageing, neurodegeneration and Parkinson's disease. *Age and Ageing*.

[107] Su X., Federoff H. J. (2014). Immune responses in Parkinson’s disease: interplay between central and peripheral immune systems. *BioMed Research International*.

[108] Schapira A. H. V., Chaudhuri K. R., Jenne P. (2017). Non-motor features of Parkinson disease. *Nature Reviews Neuroscience*.

[109] Reeve A., Simcox E., Turnbull D. (2014). Ageing and Parkinson's disease: why is advancing age the biggest risk factor?. *Ageing Research Reviews*.

[110] Xie X., Luo X., Liu N. (2017). Monocytes, microglia, and CD200-CD200R1 signaling are essential in the transmission of inflammation from the periphery to the central nervous system. *Journal of Neurochemistry*.

[111] Weiss J. M., Berman J. W. (1998). Astrocyte expression of monocyte chemoattractant protein-1 is differentially regulated by transforming growth factor beta. *Journal of Neuroimmunology*.

[112] Lindenau J. D., Altmann V., Schumacher-Schuh A. F., Rieder C. R., Hutz M. H. (2017). Tumor necrosis factor alpha polymorphisms are associated with Parkinson's disease age at onset. *Neuroscience Letters*.

[113] Perez A., Guan L., Sutherland K., Cao C. (2016). Immune system and Parkinson’s disease. *Archives of Medical Science*.

[114] Brochard V., Combadière B., Prigent A. (2009). Infiltration of CD4+ lymphocytes into the brain contributes to neurodegeneration in a mouse model of Parkinson disease. *Journal of Clinical Investigation*.

[115] Wheeler C. J., Seksenyan A., Koronyo Y. (2014). T-lymphocyte deficiency exacerbates behavioral deficits in the 6-OHDA unilateral lesion rat model for Parkinson's disease. *Journal of Neurology & Neurophysiology*.

[116] Baba Y., Kuroiwa A., Uitti R. J., Wszolek Z. K., Yamada T. (2005). Alterations of T-lymphocyte populations in Parkinson disease. *Parkinsonism and Related Disorders*.

[117] Chen L., Mo M., Li G. (2016). The biomarkers of immune dysregulation and inflammation response in Parkinson disease. *Translational Neurodegeneration*.

[118] Reale M., Iarlori C., Thomas A. (2009). Peripheral cytokines profile in Parkinson's disease. *Brain, Behavior, and Immunity*.

[119] Scalzo P., de Miranda A. S., Guerra Amaral D. C., de Carvalho Vilela M., Cardoso F., Teixeira A. L. (2011). Serum levels of chemokines in Parkinson's disease. *Neuroimmunomodulation*.

[120] Wang L., Xie Y., Zhu L. J., Chang T. T., Mao Y. Q., Li J. (2010). An association between immunosenescence and CD4^+^CD25^+^ regulatory T cells: a systematic review. *Biomedical and Environmental Sciences*.

[121] Tofaris G. K. (2017). A critical assessment of exosomes in the pathogenesis and stratification of Parkinson's disease. *Journal of Parkinson's Disease*.

[122] Tomlinson P. R., Zheng Y., Fischer R. (2015). Identification of distinct circulating exosomes in Parkinson's disease. *Annals of Clinical Translational Neurology*.

[123] Candore G., Balistreri C. R., Colonna-Romano G. (2008). Immunosenescence and anti-immunosenescence therapies: the case of probiotics. *Rejuvenation Research*.

[124] Akbari E., Asemi Z., Daneshvar Kakhaki R. (2016). Effect of probiotic supplementation on cognitive function and metabolic status in Alzheimer's disease: a randomized, double-blind and controlled trial. *Frontiers in Aging Neuroscience*.

[125] Fontana L., Partridge L. (2015). Promoting health and longevity through diet: from model organisms to humans. *Cell*.

